# Supplementary material for: Systematic Review and Critical Evaluation of Quality of Clinical Practice Guidelines on Nutrition in Pregnancy
Source: Healthcare (Basel). 2022 Dec 9;10(12):2490. doi: 10.3390/healthcare10122490 (PMC9778102; doi:10.3390/healthcare10122490)
Supplement: Supplementary file 1 [file healthcare-10-02490-s001.zip › Supplementar Table S2.pdf]

Supplementary Table 2: Critical evaluation of different topics on nutrition in pregnancy analyzed among different CPG

Issue<br>*Guidelines tot = 18 (100%)*	**N (%)**
**Scope**	
• International	3/18 (16,7%)
• National	14/18 (77,8%)
• Local	1/18 (5,5%)
**Method of developement**	
• GRADE and GRADE-CERQual approaches, DECIDE framework	1/18 (5,5%)
• Review of literature, expert panel consensus	5/18 (27,8%)
• Review of literature,expert opinion	12/18 (66,7%)
**Vitamin A**	
• 800 mcg	4/18 (22%)
• 700-750 mcg	3/18 (16,7%)
• Not stated	9/18 (50%)
• No	2/18 (11,1%)
**Vitamin C**	
• 55 mg	2/18 (11,1%)
• 70 mg	2/18 (11,1%)
• 85 mg	1/18 (5%)
• 500 mg-1000 mg	1/18(5%)
• Not stated	12/18 (66,7%)
• No	0/18 (0%)
**Vitamin D**	
• 600 mcg/die	1/18 (5%)
• 400 mcg/die	4/18 (22%)
• < o =10 mcg	3/18 (16,7%)
• <o = 5 mcg	5/18 (27,8%)
• 15 mcg	1/18 (5%)
• < o = 50 mcg	1/18 (5%)
• Not stated	3/18 (16,7%)
**Vitamin E**	
• < 10 mg	1/18 (5%)
• 10 mg	2/18 (11,1%)
• 10-30 mg	3/18 (16,7%)
• 30-60 mg	2/18 (11,1%)
• 60-120 mg	1/18 (5%)
• 200 mg	1/18 (5%)
• Not stated	8/18 (44,4%)
**Folic acid**	
• 200 mcg	5/18 (27,8%)
• 400 mcg	7/18 (38,8%)
• 600 mcg	3/18 (16,7%)
• 400-600 mcg	1/18 (5%)
• 400-800 mcg	1/18 (5%)
• Not stated	1/18 (5%)
**Iron**	
• 30-60 mg	3/18 (16,7%)
• < o = 30 mg	7/18 (38,8%)
• 60 mg	2/18 (11,1%)
• No	1/18 (5%)
• Not stated	5/18 (27,8%)
**Iodine**	
• 150 mcg	3/18 (16,7%)
• 200 mcg	1/18(5%)
• >200 mcg	3/18(16,7%)
• 1,3 mg	1/18(5%)
• 1,7 mg	1/18(5%)

• No	1/18(5%)
• Not stated	8/18 (44,4%)
**Vitamin B1**	
• 1,4 mg	4/18(22%)
• 18 mg	1/18(5%)
• 10 mg	1/18(5%)
• No	1/18(5%)
• Not stated	11/18 (61,1%)
**Vitamin B2**	
• 1,4 mg	4/18(22%)
• 1,9 mg	1/18(5%)
• No	1/18(5%)
• Not stated	12/18(66,7%)
**Vitamin B6**	
• 1,9 mg	6/18 (33,3%)
• 2,6 mg	2/18 (11,1%)
• No	0/18 (0%)
• Not stated	8/18 (44,4%)
**Vitamin B12**	
• 2,6 mcg	5/18 (27,8%)
• 2,4 mcg	1/18 (5%)
• 1,0 mcg	1/18(5%)
• 80 mg	1/18(5%)
• No	1/18(5%)
• Not stated	9/18 (50%)
**Calcium**	
• 1000-1300 mg	2 /18(11,1%)
• 1000 mg	5/18(27,8%)
• 600 mg	1/18(5%)
• 400 mg	2/18(11,1%)
• 5 mcg	1/18(5%)
• No	1/18(5%)
• Not stated	6/18 (33,3%)
**Selenium**	
• 60-65 mcg	4/18(22%)
• 30mcg	1/18(5%)
• 700 mg	1/18(5%)
• No	1/18(5%)
• Not stated	11/18 (61,1%)
**Zinc**	
• 5 mg	1/18(5%)
• 5-15 mg	5/18(27,8%)
• 205 mg	1/18(5%)
• No	1/18(5%)
• Not stated	10/18 (55,6%)
**Copper**	

• 1,15 mg	1/18(5%)
• 2 mg	2/18(11,1%)
• 8 mg	1/18(5%)
• 1000 mg	2/18(11,1%)
• 800 mg	1/18(5%)
• 1200-2000 mg	2/18(11,1%)
• No	1/18(5%)
• Not stated	8/18 (44,4%)
**Biotin**	
• 30 mcg	2/18(11,1%)
• 35 mcg	1/18(5%)
• No	1/18(5%)
• Not stated	14/18 (77,8%)
**Choline**	
• 450 mg	2/18(11,1%)
• 415 mg	1/18(5%)
• 6 mg	1/18(5%)
• No	1/18(5%)
• Not stated	13/18(72,2%)
**Carbohydrates**	
• 175 gr	3/18(16,7%)
• No	1/18(5%)
• Not stated	14/18(77,8%)
**Fibers**	
• 30-35 gr	1/18(5%)
• 28 gr	1/18(5%)
• No	0/18 (0%)
• Not stated	16/18 (88,9%)
**Protein**	
• Additional 1gr/day in 1st trimester; additional 9gr/day in 2nd trimester; additional ≤ 28gr/day or one extra serving from food pyramid	1/18(5%)
• 0.8-1.0gr/kg in the first half of pregnancy, 1.1 gr/kg in the second half	1/18(5%)
• 450 mg/die	1/18(5%)
• 71 gr	1/18(5%)
• 60 gr	1/18(5%)
• 10 gr	1/18(5%)
• No	0/18 (0%)
• Not stated	12/18 (66,7%)
**Fat**	
• Omega 3- Omega 6	1/18(5%)
• 200-300 mg of dochosahexaenoic acid	1/18(5%)
• Additional 700-1400 mg/week DHA	1/18(5%)

• >175 g	1/18(5%)
• No	0/18 (0%)
• Not stated	14/18(77,8%)
**Omega-3**	
• 28g/die	1/18(5%)
• 200 mg DHA	1/18(5%)
• 700-1400 mg/week	1/18(5%)
• 1 g	1/18(5%)
• 300 mg	1/18(5%)
• 800 mg	1/18(5%)
• 1.4 gr	1/18(5%)
• No	0/18 (0%)
• Not stated	11/18 (61,1%)
**Omega 6**	
• aumentare 1g/die nel I trimestre, 8g/die nel II e 23-26g/die nel III trimestre	2/18(11,1%)
• 10 gr	1/18(5%)
• 13 gr	1/18(5%)
• No	0/18(0%)
• Not stated	14/18(77,8%)
**Mercury**	
• 250 mg/die	1/18(5%)
• 150mg di pesce ricco in omega-3 /settimana	1/18(5%)
• No	0/18(0%)
• Not stated	16/18(88,9%)
**Hydration**	
• 2-2.5 L of water	3/18(16,7%)
• No	0/18(0%)
• Not stated	15/18(83,3%)
**Alcohol**	
• No	4/18(22,2%)
• Yes	1/18(5%)
**Weigh gain in pregnancy**	13/18 (72,2%)
• BMI < 18,5: 12,5-18,0 kg BMI 18,5-25 : 11,5 - 16,0 kg BMI 25-30: 7,0-11,5 kg BMI≥ 30: 5,0-9,0 kg	3/18(16,7%)
• BMI < 18,5: 12,5-18,0 kg BMI 18,5-25 : 11,5 - 16,0 kg BMI 25-30: 7,0-11,5 kg BMI≥ 30: ≤6.8	1/18(5%)
• BMI≥ 30: ≤6	1/18 (5%)
• Yes	2/18 (11,1%)
• No	0/18(0%)
**Prevention of food-borne diseases in pregnancy**	10/18(55,5%)
• • Listeria, Salmonella	1/18(5%)
• Listeria, Salmonella, Toxoplasma	1/18(5%)

• Listeria, Salmonella, Toxoplasma, Environmental contaminants	1/18(5%)
• Preventive anthelminthic treatment after first trimester	1/18(5%)
• Yes	1/18(5%)
• No	0/18 (0%)
• Not specified	13/18 (72,2%)
**Nutrition in particular groups of pregnant women**	
• Adolescent, Multiple Pregnancy,Vegan and vegetarian Women	1/18(5%)
• Multifetal pregnancy	1/18(5%)
• Yes	1/18(5%)
• No	0/18(0%)
• Not specified	15/18 (83,3%)
**Maternal outcomes in association with optimal nutrition**	
• Yes	5/18(27,8%)
• No	0/18(0%)
• Not specified	13/18(72,2
**Fetal/Neonatal outcomes (SGA)**	
• Yes	7/18(38,9%)
• no	0/18(0%)
• Not specified	11/18(61,1%)
**Fetal/Neonatal outcomes (Low birthweight)**	
• Yes	5/18(27,8%)
• No	0/18(0%)
• Not specified	13/18(72,2%)
**Fetal/Neonatal outcomes (Preterm Birth)**	
• Yes	5/18(27,8%)
• No	0/18(0%)
• Not specified	13/18(72,2%)
**Fetal/Neonatal outcomes(Perinatal mortality)**	
• Yes	1/18 (5%)
• No	1/18(5%)
• Not specified	16/18(88,9%)
**Fetal/Neonatal outcomes(Neonatal mortality)**	
• Yes	3/18(16,6%)
• No	0/18(0%)
• Not specified	15/18(83,3%)
**Fetal/Neonatal outcomes(Stillbirth)**	
• Yes	3/18(16,7%)
• No	0/18(0%)
• Not specified	15/18(83,3%)
**Fetal/Neonatal outcomes Congenital anomalies**	
• Yes	3/18(16,7%)
• No	0/18(0%)
• Not specified	15/18(83,3%)
